# Changes in Incident Schizophrenia Diagnoses Associated With Cannabis Use Disorder After Cannabis Legalization

**DOI:** 10.1001/jamanetworkopen.2024.57868

**Published:** 2025-02-04

**Authors:** Daniel T. Myran, Michael Pugliese, Lyndsay D. Harrison, Marco Solmi, Kelly K. Anderson, Jess G. Fiedorowicz, Yaron Finkelstein, Doug Manuel, Monica Taljaard, Colleen Webber, Peter Tanuseputro

**Affiliations:** 1 Ottawa Hospital Research Institute, Ottawa, Ontario, Canada; 2 Department of Family Medicine, University of Ottawa, Ottawa, Ontario, Canada; 3 ICES uOttawa, Ottawa Hospital Research Institute, Ottawa, Ontario, Canada; 4 Bruyère Health Research Institute, Ottawa, Ontario, Canada; 5 Department of Psychiatry, University of Ottawa, Ottawa, Ontario, Canada.; 6 Department of Mental Health, The Ottawa Hospital, Ottawa, Ontario, Canada.; 7 School of Epidemiology and Public Health, Faculty of Medicine, University of Ottawa, Ottawa, Ontario, Canada; 8 Department of Child and Adolescent Psychiatry, Charité Universitätsmedizin, Berlin, Germany; 9 Department of Epidemiology and Biostatistics, Schulich School of Medicine & Dentistry, Western University, London, Ontario, Canada; 10 Department of Psychiatry, Schulich School of Medicine & Dentistry, Western University, London, Ontario, Canada; 11 ICES Western, London, Ontario, Canada; 12 Division of Pediatric Emergency Medicine, Hospital for Sick Children, Toronto, Ontario, Canada; 13 Division of Clinical Pharmacology and Toxicology, Hospital for Sick Children, Toronto, Ontario, Canada; 14 Department of Paediatrics, University of Toronto, Toronto, Ontario, Canada; 15 Department of Pharmacology and Toxicology, University of Toronto, Toronto, Ontario, Canada

## Abstract

**Importance:**

Despite public health concerns that cannabis legalization may increase the number of cases of schizophrenia caused by cannabis, there is limited evidence on this topic.

**Objective:**

To examine changes in the population-attributable risk fraction (PARF) for cannabis use disorder (CUD) associated with schizophrenia after liberalization of medical cannabis and legalization of nonmedical cannabis in Canada.

**Design, Setting, and Participants:**

This population-based cohort study was conducted in Ontario, Canada, from January 1, 2006, to December 31, 2022, among 13 588 681 people aged 14 to 65 years without a history of schizophrenia.

**Exposures:**

Diagnosis of CUD in the emergency department or hospital setting (*International Statistical Classification of Diseases and Related Health Problems, Tenth Revision, Canada* [*ICD-10-CA*] codes F12x and T40.7).

**Main Outcome and Measures:**

Changes in the PARF for CUD associated with schizophrenia (*ICD-10-CA* codes F20x and F25x and *Diagnostic and Statistical Manual of Mental Disorders* [Fourth Edition] [*DSM-IV*] code 295x) over 3 policy periods: prelegalization (January 2006 to November 2015), liberalization of medical and nonmedical cannabis (December 2015 to September 2018), and legalization of nonmedical cannabis (October 2018 to December 2022). A secondary outcome was diagnosis of psychosis not otherwise specified (NOS) (*ICD-10-CA* code F29x and *DSM-IV* code 298x). Segmented linear regression was used to examine changes after the liberalization of medical cannabis in 2015 and the legalization of nonmedical cannabis in 2018.

**Results:**

The study included 13 588 681 individuals (mean [SD] age, 39.3 [16.1] years; 6 804 906 males [50.1%]), of whom 118 650 (0.9%) had CUD. A total of 91 106 individuals (0.7%) developed schizophrenia (80 523 of 13 470 031 [0.6%] in the general population without CUD vs 10 583 of 118 650 [8.9%] with CUD). The PARF for CUD associated with schizophrenia almost tripled from 3.7% (95% CI, 2.7%-4.7%) during the prelegalization period to 10.3% (95% CI, 8.9%-11.7%) during the legalization period. The PARF in the postlegalization period ranged from 18.9% (95% CI, 16.8%-21.0%) among males aged 19 to 24 years to 1.8% (95% CI, 1.1%-2.6%) among females aged 45 to 65 years. The annual incidence of schizophrenia was stable over time, while the incidence of psychosis NOS increased from 30.0 to 55.1 per 100 000 individuals (83.7%) in the postlegalization period relative to the prelegalization period. The PARF for CUD associated with schizophrenia increased steadily over the study with no accelerations after cannabis policy changes, while increases in the PARF for CUD associated with psychosis NOS accelerated after medical cannabis liberalization.

**Conclusions and Relevance:**

In this cohort study of individuals aged 14 to 65 years in Ontario, Canada, the proportion of incident cases of schizophrenia associated with CUD almost tripled during a period of substantial liberalization of cannabis policy. Ongoing research is indicated to understand the long-term associations of cannabis policy with the prevalence of psychotic disorders.

## Key Points

**Question:**

Were the liberalization of medical cannabis and the legalization of nonmedical cannabis in Canada associated with changes in the population-attributable fraction of cannabis use disorders associated with schizophrenia?

**Findings:**

In this population-based cohort study comprising 13 588 681 individuals, the population-attributable fraction of cannabis use disorder associated with schizophrenia increased significantly from 3.7% in the prelegalization period to 10.3% during the postlegalization period.

**Meaning:**

These findings suggest that the association between cannabis use disorders and schizophrenia is an important consideration for the legalization of cannabis.

## Teaser

This cohort study of more than 13 million individuals aged 14 to 65 years examines changes in the population-attributable risk fraction for cannabis use disorder associated with schizophrenia after liberalization of medical cannabis and legalization of nonmedical cannabis in Canada.

## Introduction

Cannabis use is associated with subsequent development or earlier onset of psychosis and schizophrenia.^1,2,3,4,5,6,7^ Evidence suggests a dose-response relationship, with more frequent use of higher-potency cannabis products associated with a greater risk of schizophrenia.^8^ Cannabis use and the tetrahydrocannabinol (THC) content of cannabis products are increasing globally.^8,9^ A recent study estimated that the proportion of cases of schizophrenia in Denmark associated with cannabis increased from approximately 4% in 2000 to 8% from 2010 to 2016.^10^ However, cannabis was and remains illegal in Denmark and a frequently raised public health concern is that legalization may result in further increases in cannabis use and the incidence of psychotic disorders.^10^ Evidence on the association between cannabis legalization and psychosis is lacking. Studies from Canada and the US examining periods immediately after cannabis legalization found no significant associations between legalization and increases in psychotic disorders.^11,12^ However, prior studies have been underpowered to detect changes in psychotic disorders given that they are relatively rare and that there are anticipatable multiple-year lags between cannabis policy and detection of potential changes in psychotic disorders. Mechanistically, legalization would gradually increase cannabis use, increasing cannabis use would increase the incidence of psychosis, and individuals with incident psychosis would then need to be diagnosed in the health system. Consequently, studies evaluating changes in the proportion of incident cases of schizophrenia associated with cannabis use after policy changes can provide an earlier indicator of the possible associations of legalization with schizophrenia.

Cannabis policy in Canada has shifted markedly over the past 2 decades. Medical cannabis in Canada has been legal since 2001 for a limited list of severe or chronic medical conditions and was greatly expanded in 2014 for anyone with medical authorization from a physician indicating they would therapeutically benefit from cannabis.^13^ In December 2015, the federal government committed to legalizing nonmedical cannabis; after this announcement, there were large increases in illicit and gray market medical and nonmedical cannabis dispensaries and online vendors.^14^ Legalization of nonmedical cannabis came into effect in October 2018, making Canada the first country in the world to allow commercial sale of nonmedical cannabis.^15,16^ The current study is situated in Ontario, Canada’s most populous province (14.5 million residents). In Ontario, there were initially restrictions on the number of legal cannabis stores and types of products that could come to market.^17,18^ In early 2020, the market in Ontario began to commercialize with the introduction of new products with high THC content (concentrates, vapes, and edibles) along with the removal of store restrictions.^17,18^

These policy changes, combined with robust health administrative data capturing all health system encounters for all residents of Ontario, provide a unique opportunity to understand the association of cannabis policy changes with the risk of schizophrenia. We examined how the population-attributable risk fraction (PARF) for cannabis use disorder (CUD) associated with schizophrenia changed over time in Ontario in response to medical cannabis liberalization and nonmedical cannabis legalization with restrictions. PARFs estimate the proportion of cases of a condition that could have been prevented if the exposure were eliminated, assuming a causal relationship between exposure and outcome—in our study, the cases of schizophrenia that could have been prevented if cannabis use patterns severe enough to require care in the emergency department (ED) or hospital were eliminated.^19,20^ We also examined differences in changes over time stratified by age and sex.

## Methods

This project was authorized under section 45 of Ontario’s Personal Health Information Protection Act, which authorizes ICES (formerly known as the Institute for Clinical Evaluative Sciences) to collect personal health information without consent for health system management, evaluation, monitoring, or planning. It was approved by ICES Privacy Office. No informed consent was required as the study used deidentified health information. This study followed the Strengthening the Reporting of Observational Studies in Epidemiology (STROBE) reporting guideline. Our study protocol was prespecified and registered with the open-science registries network.^21^

### Study Design, Population, and Data Sources

We conducted a population-based retrospective cohort study examining annual changes in the PARF of schizophrenia spectrum disorder (herein referred to as *schizophrenia*) associated with CUD between January 1, 2006, and December 31, 2022. We used routinely collected health administrative data from the province of Ontario, Canada. We included all Ontario residents aged 14 to 65 years who were eligible for the province’s single-payer universal health system (the Ontario Health Insurance Plan [OHIP], which covers an estimated 97% of residents of Ontario) in at least 1 study year. We selected the upper age limit of 65 years to minimize misclassification of schizophrenia with dementia.^22^ Each year, we excluded people who had not been continually eligible for OHIP in the previous 3 years, to ensure capture of our primary exposure, and excluded individuals who had already received a diagnosis of schizophrenia, defined as health service contact for schizophrenia in the previous 10 years. We used an interrupted time series design to examine changes in the PARF after specific changes in cannabis policy. Data on all ED visits, hospitalizations, and outpatient physician visits and sociodemographic characteristics were obtained using 7 individual-level datasets. These datasets were linked using unique encoded identifiers and analyzed at ICES (see eMethods 1 in Supplement 1 for details on database holdings).

### Exposures

#### Cannabis Use Disorder

We identified individuals with 1 or more ED visits or inpatient hospitalizations for CUD, defined when an *International Statistical Classification of Diseases and Related Health Problems, Tenth Revision, Canada* (*ICD-10-CA*) code for cannabis use (F12.X [mental and behavioral disorders due to the use of cannabinoids] or T40.7 [poisoning by cannabis, including derivatives]) were listed as the main or contributing reason for the ED visit or as the primary or contributing diagnosis from a hospital admission on discharge. Attribution of diagnostic codes as main or primary or contributing reason or diagnosis is based on the clinical judgment of the treating team. In April 2019 specialized mental health beds in Ontario migrated from using *International Classification of Diseases, Ninth Revision, Clinical Modification* (*ICD-9-CM*) to *ICD-10-CM* coding. Before April 2019, we used *ICD-9-CM* codes 304.30 (cannabis dependence) and 305.20 (cannabis abuse) to identify hospitalizations due to cannabis.^23^ We additionally captured hospitalization due to cannabis in specialized mental health beds when 1 of the following *International Classification of Diseases, Ninth Revision*, codes was listed as a main or contributing reason for hospitalizations: 304.30 (cannabis dependence) and 305.20 (cannabis abuse).^23^

#### Cannabis Policy

We considered the following policy periods: prelegalization (January 2006 to November 2015), liberalization of medical and nonmedical cannabis (December 2015 to September 2018), and legalization of nonmedical cannabis (October 2018 to December 2022). In previous work, the legalization period was divided into legalization with restrictions (October 2018 to February 2020) and cannabis commercialization (March 2020 onward).^24^ We chose a priori to examine an overall legalization period due to the length of the restricted legalization period and the lag in our outcome.

### Outcomes

#### Primary Outcome

We identified the first onset of schizophrenia based on a medical record–validated algorithm (sensitivity, 91.6%; specificity, 61.3%).^25^ Schizophrenia was defined when a diagnostic code for schizophrenia or schizoaffective disorder (*ICD-10-CM* codes F20x or F25x and *DSM-IV* code 295x) was the (1) primary discharge diagnosis from a general hospital bed, (2) a discharge diagnosis from a psychiatric hospital bed, or (3) the main or contributing reason for 2 or more outpatient visits or ED visits occurring within 12 months of each other (with the date of first onset at the time of the second visit) (eMethods 2 in Supplement 1).

#### Secondary Outcome

We used the primary outcome schizophrenia definition and additionally included codes for psychotic disorder not otherwise specified (NOS) (*ICD-10-CM* code F29x and *DSM-IV* code 298x). For this secondary outcome, each year, we further excluded individuals with health service contact for psychosis NOS in the previous 10 years.

### Covariates

We obtained demographic details, including age, sex, rural residence, neighborhood income quintile, immigration status, and health care use in the past 3 years, including outpatient mental health visits and ED visits and hospitalizations for substance use and mental health conditions. See eMethods 3 in Supplement 1 for covariate details.^23^

### Statistical Analysis

We described annual changes in key indicators, including incident schizophrenia diagnoses and prevalence of CUD. We then used the 2022 population of Ontario as a reference to generate age, sex, income, rurality, and immigration status standardized annual rates. We then calculated changes in the PARF over time. We used the following formula to calculate the PARF: PARF = pd (AHR − 1) / AHR, where pd is the proportion of exposed participants in cases and AHR is the adjusted hazard ratio obtained from our modeling approach. This method is considered internally valid in the presence of confounding, assuming all relevant confounders have been identified and adjusted.^19,20^ Hazard ratios (HRs) can be used in the calculation of PARFs when the outcome is uncommon.^19^ Given established differences in age of onset of schizophrenia between males and females, we completed 8 stratified analyses for males and females separately at the ages of 14 to 18 years, 19 to 24 years, 25 to 44 years, and 45 to 65 years.^26,27^

We obtained HRs for the PARF calculations using multivariable cause-specific hazard models accounting for the competing risk of all-cause mortality. Each year in the study, we completed an individual-level analysis to identify a year-specific HR for the association between CUD and incident cases of schizophrenia or psychosis NOS. Individuals entered the analysis on January 1 of each year and were censored on December 31 of each year if they had not developed schizophrenia, died, or lost OHIP eligibility. Exposure was treated as a time-varying covariate in analyses where each year, individuals had their exposure established on January 1 (exposed if they had 1 ED visit or hospitalization involving cannabis on or in the 3 years) and unexposed individuals could become exposed between January 1 and being censored. We adjusted the hazard models for age (splines at the 5th, 27.5th, 50th, 72.5th, and 95th percentiles), sex (male or female), income quintile (with a sixth category for missing), rural residence (urban or rural or missing), immigration status, past 3-year ED visits or hospitalizations for mental health (yes or no separately for mood, anxiety, self-harm, and other), past 3-year substance use ED visits or hospitalizations (yes or no separately for alcohol, opioids, and other), and past 3-year outpatient mental health care visits (yes or no separately for family medicine and psychiatry). We combined data from each year together into our full dataset for presentation.

We completed 2 sensitivity analyses for the PARF calculators. First, we generated HRs without adjusting for past 3-year mental health or past 3-year substance use, which may be mediators rather than confounders. Second, we used an unlimited lookback period (until database inception in 1991) when excluding prior cases of schizophrenia.

We calculated PARFs for each of the 3 policy periods: (1) prelegalization, (2) liberalization of medical cannabis and announcement of nonmedical legalization, and (3) nonmedical cannabis legalization. We used linear segmented regression to examine changes in the quarterly PARF after each policy change. Given that the PARF is based on past 3-year health care visits, we examined only gradual (slope) changes rather than gradual and immediate changes. All analyses included first-order autocorrelation and adjusted for seasonality. We expressed the gradual change after each policy period as rate changes with 95% CIs. All statistical analyses were completed using SAS Enterprise Guide, version 7.1 (SAS Institute Inc). Statistical significance was determined by 95% CIs that did not cross 1.

## Results

Over the 17-year study, we included 13 588 681 individuals (mean [SD] age, 39.3 [16.1] years; 6 804 906 males [50.1%] and 6 783 775 females [49.9%]) in 1 or more years of analysis, of whom 118 650 (0.9%) had an ED visit or hospitalization for a CUD (see the eFigure in Supplement 1 for a flowchart of cohort exclusions). Over the study period, 91 106 (0.7%) individuals developed schizophrenia: 10 583 of 118 650 individuals with CUD (8.9%) developed schizophrenia, compared with 80 523 of 13 470 031 individuals without CUD (0.6%) (Table 1). Compared with individuals without CUD, individuals with CUD were more likely to be male (72 418 of 118 650 [61.0%] vs 6 732 488 of 13 470 031 [50.0%]), to be younger (mean [SD] age, 27.5 [12.1] years vs 39.4 [16.1] years), and to live in lower-income neighborhoods (33 799 of 118 650 [28.5%] vs 2 655 333 of 13 470 031 [19.7%] in the lowest income quintile) and less likely to be immigrants to Canada (10 475 of 118 650 [8.8%] vs 2 877 440 of 13 470 031 [21.4%]) Individuals with CUD had substantially higher levels of prior outpatient and acute care associated with mental health and substance use.

**Table 1.** Characteristics of Individuals Included in at Least 1 Year of Study

Characteristic	No. (%)		
	ED visit or hospitalization for cannabis use disorder (n = 118 650)	General population (n = 13 470 031)	Total population (N = 13 588 681)
Sex			
Male	72 418 (61.0)	6 732 488 (50.0)	6 804 906 (50.1)
Female	46 232 (39.0)	6 737 543 (50.0)	6 783 775 (49.9)
Age, y			
Mean (SD)	27.5 (12.1)	39.4 (16.1)	39.3 (16.1)
14-18	33 454 (28.2)	1 902 932 (14.1)	1 936 386 (14.2)
19-24	29 402 (24.8)	1 400 833 (10.4)	1 430 235 (10.5)
25-44	41 248 (34.8)	4 588 941 (34.1)	4 630 189 (34.1)
45-65	14 546 (12.3)	5 577 325 (41.4)	5 591 871 (41.2)
Rurality			
Urban	103 890 (87.6)	12 077 044 (89.7)	12 180 934 (89.6)
Rural	14 242 (12.0)	1 369 832 (10.2)	1 384 074 (10.2)
Neighborhood income quintile			
1 (Lowest)	33 799 (28.5)	2 655 333 (19.7)	2 689 132 (19.8)
2	24 908 (21.0)	2 640 110 (19.6)	2 665 018 (19.6)
3	21 570 (18.2)	2 669 323 (19.8)	2 690 893 (19.8)
4	19 746 (16.6)	2 716 296 (20.2)	2 736 042 (20.1)
5 (Highest)	17 810 (15.0)	2 741 000 (20.3)	2 758 810 (20.3)
Immigration status			
Born outside Canada	10 475 (8.8)	2 877 440 (21.4)	2 887 915 (21.3)
Substance use acute care visits in past 3 y			
Alcohol	14 963 (12.6)	127 378 (0.9)	142 341 (1.0)
Opioids	4051 (3.4)	18 299 (0.1)	22 350 (0.2)
Cocaine	5938 (5.0)	15 961 (0.1)	21 899 (0.2)
Amphetamine	2814 (2.4)	5526 (0.04)	8340 (0.1)
Polysubstance use	9382 (7.9)	32 559 (0.2)	41 941 (0.3)
Other	1948 (1.6)	5189 (0.04)	7137 (0.1)
Mental health acute care visits in past 3 y			
Any	50 725 (42.8)	477 246 (3.5)	527 971 (3.9)
Mood disorder	17 255 (14.5)	136 362 (1.0)	153 617 (1.1)
Anxiety disorder	20 470 (17.3)	226 737 (1.7)	247 207 (1.8)
Deliberate self-harm	10 672 (9.0)	69 313 (0.5)	79 985 (0.6)
Other	35 041 (29.5)	186 536 (1.4)	221 577 (1.6)
Outpatient mental health and addiction visits in past 3 y			
Any	50 092 (42.2)	2 017 699 (15.0)	2 067 791 (15.2)
Family physician	43 109 (36.3)	1 844 736 (13.7)	1 887 845 (13.9)
Psychiatrist	20 922 (17.6)	393 715 (2.9)	414 637 (3.1)
Primary outcome or death			
Developed schizophrenia	10 583 (8.9)	80 523 (0.6)	91 106 (0.7)
Died	4823 (4.1)	331 765 (2.5)	336 588 (2.5)

Abbreviation: ED, emergency department.

**Figure 1.**
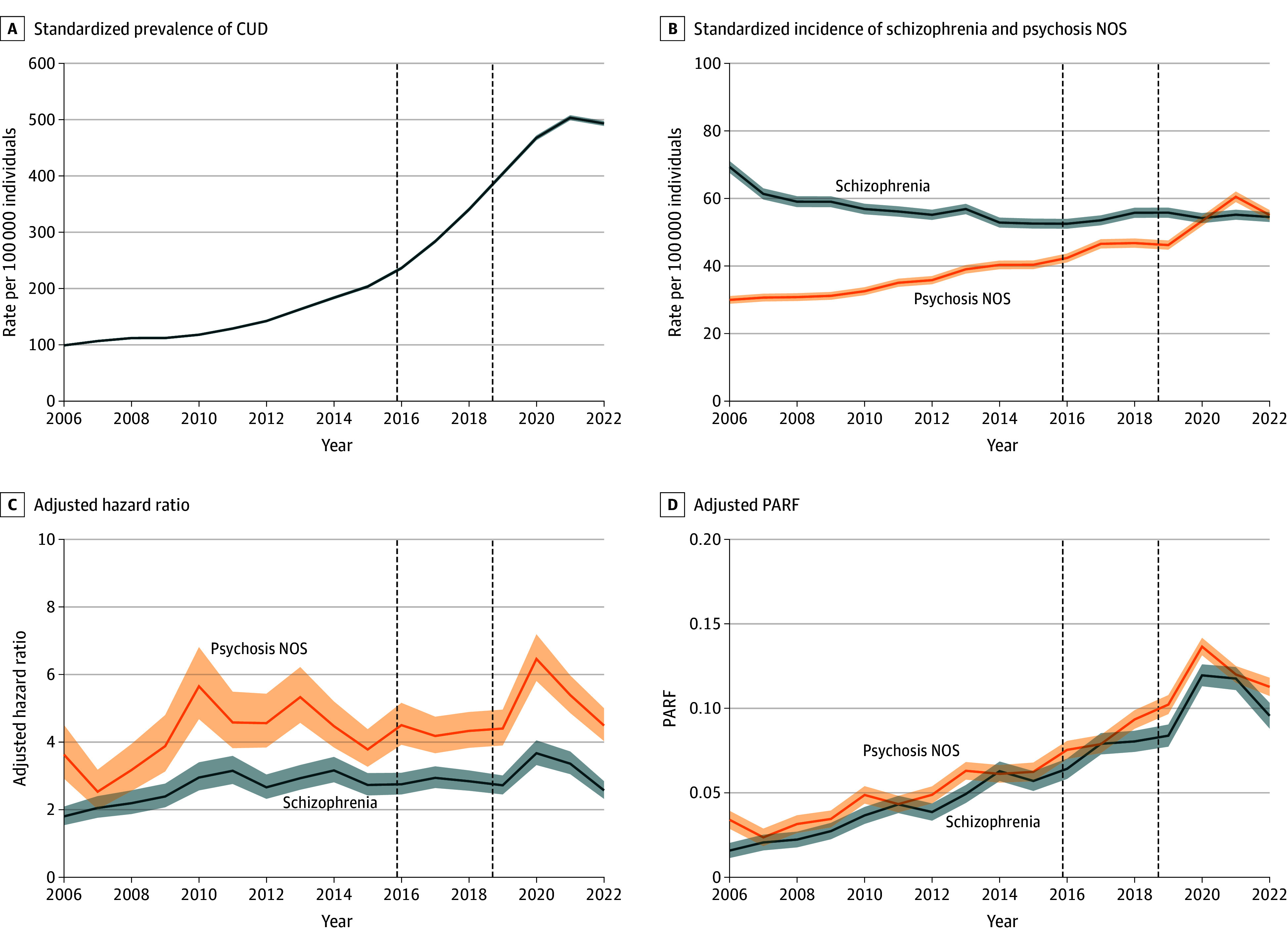
Changes in the Past 3-Year Prevalence of Cannabis Use Disorder (CUD), Incidence of Schizophrenia and Psychosis Not Otherwise Specified (NOS), and the Population-Attributable Risk Fraction (PARF) Over Time Rates are standardized for age and sex using the 2022 population as a reference. Shaded regions indicate 95% CIs. The dashed vertical lines indicate, from left to right, the liberalization of medical and nonmedical cannabis and the legalization of nonmedical cannabis, respectively.

**Figure 2.**
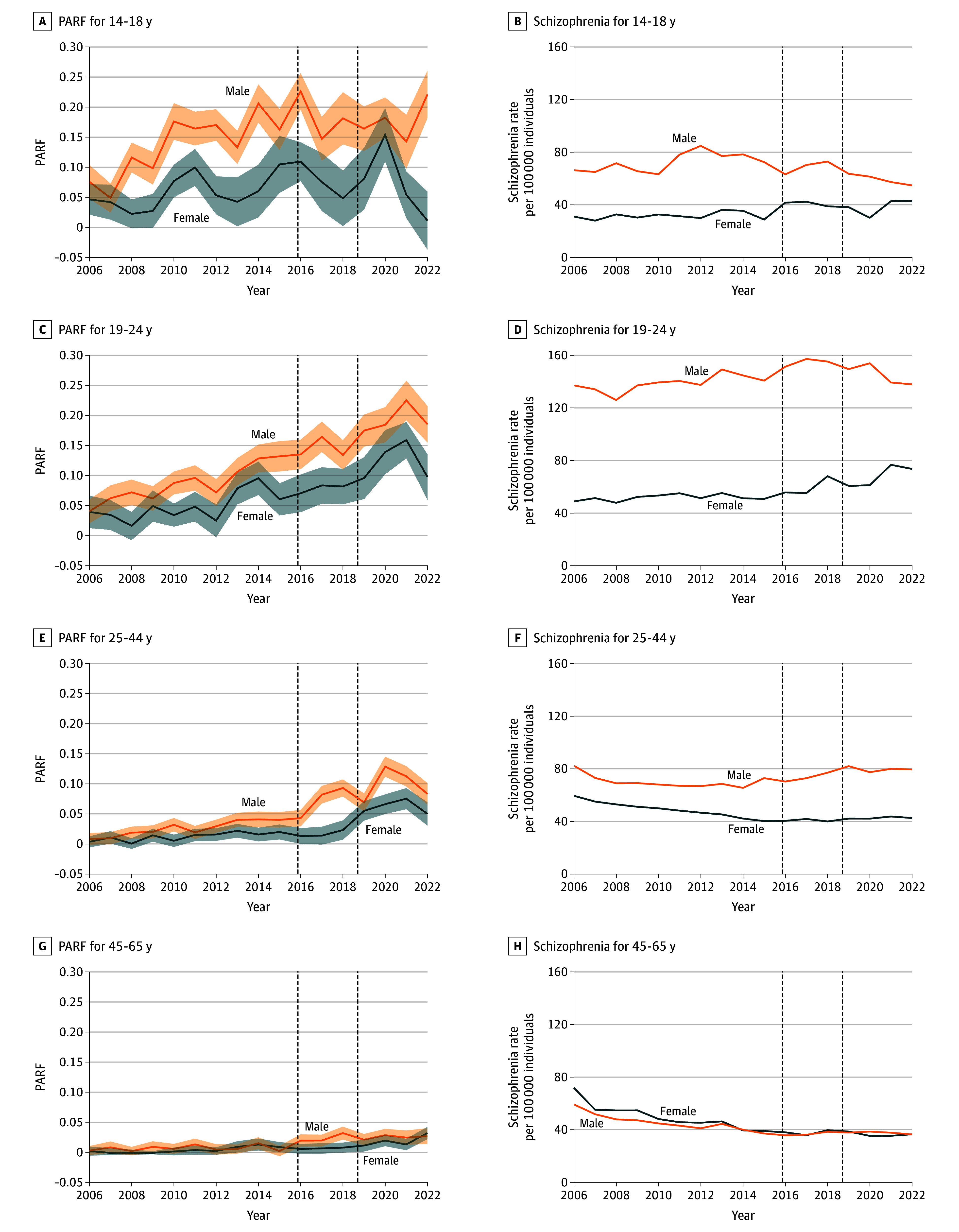
Changes in the Population-Attributable Risk Fraction (PARF) Over Time of Cannabis Use Disorder (CUD) Associated With Schizophrenia and the Incidence of Schizophrenia by Age and Sex Shaded regions indicate 95% CIs. The dashed vertical lines indicate, from left to right, the liberalization of medical and nonmedical cannabis and the legalization of nonmedical cannabis, respectively.

Figure 1 shows changes in the incidence of CUD, schizophrenia, and psychosis NOS, along with the association between CUD and schizophrenia and between CUD and psychosis NOS and the PARF over time. Between 2006 and 2022, the annual age- and sex-standardized incidence rate of CUD increased by 497.4% (from 99.2 to 493.2 per 100 000 individuals). The annual age- and sex-standardized incidence of schizophrenia decreased by 27.2% between 2006 and 2022 (from 69.3 to 54.5 per 100 000 individuals). The annual age- and sex-standardized incidence of psychosis NOS increased by 83.7% between 2006 and 2022 (from 30.0 to 55.1 per 100 000 individuals). The same trends were observed with crude rates and adjustment for changes immigration status (eTable 1 in Supplement 1). The AHR for the association between CUD in the past 3 years and development of schizophrenia ranged between 1.80 (95% CI, 1.54-2.09) in 2006 to 3.67 (95% CI, 3.32-4.05) in 2020 and varied over time. The adjusted PARF increased from 1.6% (95% CI, 1.1%-2.0%) in 2006 to 9.6% (95% CI, 8.8%-10.3%) in 2022. The adjusted PARF for CUD in the past 3 years and development of psychosis NOS increased from 3.4% (95% CI, 2.9%-3.9%) in 2006 to 11.3% (95% CI, 10.7%-11.8%) in 2022. The PARF varied by age and sex but increased over time in all age and sex groups (Figure 2). The mean (SD) age at onset of schizophrenia decreased from 38.8 (13.6) years in 2006 to 36.4 (14.1) years in 2022.

In sensitivity analyses, the estimated PARF in 2022 remained higher when not using any adjustment for past care for mental health or substance use (15.1% [95% CI, 14.8-15.5%]) than when using an unlimited lookback for excluding cases of schizophrenia (10.2% [95% CI, 9.3%-11.0%]) (see eTable 2 in Supplement 1 for values across all years). Table 2 summarizes changes in key population-level indicators of the association between CUD and schizophrenia and the burden of CUD on schizophrenia by legalization policy period. The proportion of incident cases of schizophrenia among patients who were treated for CUD in the past 3 years increased from 7.0% (3637 of 52 337) in the prelegalization period to 16.7% (3553 of 21 317) during the legalization period. The adjusted PARF of CUD associated with schizophrenia almost tripled from 3.7% (95% CI, 2.7%-4.7%) during the prelegalization period to 10.3% (95% CI, 8.9%-11.7%) during the postlegalization period.

**Table 2.** Changes in CUD, Schizophrenia, and the Population-Attributable Fraction by Cannabis Policy Period

Measure	Policy period		
	Medical cannabis with restrictions, Q1 2006 to Q3 2015	Medical and nonmedical cannabis liberalization, Q4 2015 to Q3 2018	Nonmedical legalization, Q4 2018 to Q4 2022
Age- and sex-standardized rate per 100 000 person-years			
Prevalence of CUD in past 3 y	35.4	143.3	182.4
Incident schizophrenia	53.5	52.8	53.3
Incident psychosis NOS	33.9	45.8	54.3
Incident schizophrenia, No.	52 337	14 195	21 317
Incident schizophrenia with CUD exposure, No. (%)	3637 (7.0)	1672 (11.8)	3553 (16.7)
Incident psychosis NOS, No.	35 533	12 471	21 942
Incident psychosis NOS with CUD exposure, No. (%)	2379 (6.7)	1348 (10.8)	3278 (14.9)
Adjusted HR (95% CIs) for development of schizophrenia and psychosis NOS for those with CUD vs those without CUD^a^			
CUD and schizophrenia	2.60 (2.30-2.89)	2.83 (2.75-2.92)	3.07 (2.67-3.47)
CUD and psychosis NOS	4.17 (3.58-4.76)	4.29 (4.09-4.49)	5.13 (4.38-5.89)
Adjusted PARF (95% CIs) for development of schizophrenia and psychosis NOS associated with CUD^a^			
CUD and schizophrenia	3.68 (2.72-4.65)	7.26 (6.42-8.09)	10.27 (8.86-11.68)
CUD and psychosis NOS	4.47 (3.61-5.32)	8.00 (7.13-8.86)	11.64 (10.43-12.85)
Incidence rate of schizophrenia per 100 000 person-years, by age and sex			
Male			
14-18 y	63.2	59.2	51.3
19-24 y	141.7	157.5	146.7
25-44 y	69.5	75.0	81.2
45-65 y	43.7	36.4	36.3
Female			
14-18 y	29.2	38.7	35.6
19-24 y	52.5	59.2	68.7
25-44 y	49.0	41.0	43.4
45-65 y	49.2	37.5	35.6
Adjusted PARF (95% CIs) for CUD and schizophrenia by age and sex^b^			
Male			
14-18 y	13.44 (10.37-16.50)	18.33 (15.09-21.56)	17.77 (15.24-20.30)
19-24 y	8.46 (6.67-10.25)	14.43 (13.04-15.82)	18.88 (16.79-20.97)
25-44 y	2.55 (1.79-3.30)	6.82 (4.64-9.01)	9.80 (7.75-11.86)
45-65 y	0.68 (0.41-0.96)	2.10 (1.33-2.87)	2.54 (2.26-2.83)
Female			
14-18 y	5.63 (3.95-7.30)	8.23 (5.85-10.61)	7.33 (2.76-11.91)
19-24 y	4.77 (3.27-6.28)	7.68 (6.94-8.42)	12.04 (9.50-14.57)
25-44 y	1.21 (0.78-1.64)	1.65 (1.23-2.06)	5.94 (4.75-7.13)
45-65 y	0.34 (0.04-0.64)	0.65 (0.56-0.75)	1.81 (1.06-2.56)

Abbreviations: CUD, cannabis use disorder; HR, hazard ratio; NOS, not otherwise specified; PARF, population-attributable risk fraction; Q, quarter.

^a^ Adjusted for age in splines, sex, income quintile, rural location, immigration status, past 3-year acute mental health care (separately for mood, anxiety, self-harm, other), past 3-year substance use health care (separately for alcohol, opioids, other), and past 3-year outpatient mental health care (separately for family medicine and psychiatry).

^b^ Adjusted for income quintile, rural location, immigration status, past 3-year acute mental health care (separately for mood, anxiety, self-harm, other), past 3-year substance use health care (separately for alcohol, opioids, other), and past 3-year outpatient mental health care (separately for family medicine and psychiatry).

The overall incidence of schizophrenia per 100 000 individuals was stable in the prelegalization and postlegalization periods (prelegalization, 53.5; postlegalization, 53.3) while the incidence of psychosis NOS increased by 60.2% (prelegalization, 33.9; postlegalization, 54.3) (Table 2). The annual incidence rate of schizophrenia per 100 000 individuals increased from 29.2 to 35.6 (21.8%) among females aged 14 to 18 years and from 52.5 to 68.7 (30.9%) among females aged 19 to 24 years while declining from 49.0 to 43.4 (11.3%) among females aged 25 to 44 years and from 49.2 to 35.6 (27.7%) among females aged 45 to 65 years. The annual incidence rate of schizophrenia per 100 000 individuals decreased from 63.2 to 51.3 (18.8%) among males aged 14 to 18 years and from 43.7 to 36.6 (16.9%) among males aged 45 to 65 years while increasing from 141.7 to 146.7 (3.5%) among males aged 19 to 24 years and from 69.5 to 81.2 (16.8%) among males aged 25 to 44 years. During the legalization period, the PARF was highest among males aged 19 to 24 years (18.9% [95% CI, 16.8%-21.0%]) and males aged 14 to 18 years (17.8% [95% CI, 15.2%-20.3%]) and lowest among females aged 45 to 65 years (1.8% [95% CI, 1.1%-2.6%]). The largest absolute increase in PARF between the prelegalization and postlegalization periods was among individuals aged 19 to 24 years, which increased among males from 8.5% (95% CI, 6.7%-10.3%) to 18.9% (95% CI, 16.8%-21.0%) and among females from 4.8% (95% CI, 3.3%-6.3%) to 12.0% (95% CI, 9.5%-14.6%).

**Figure 3.**
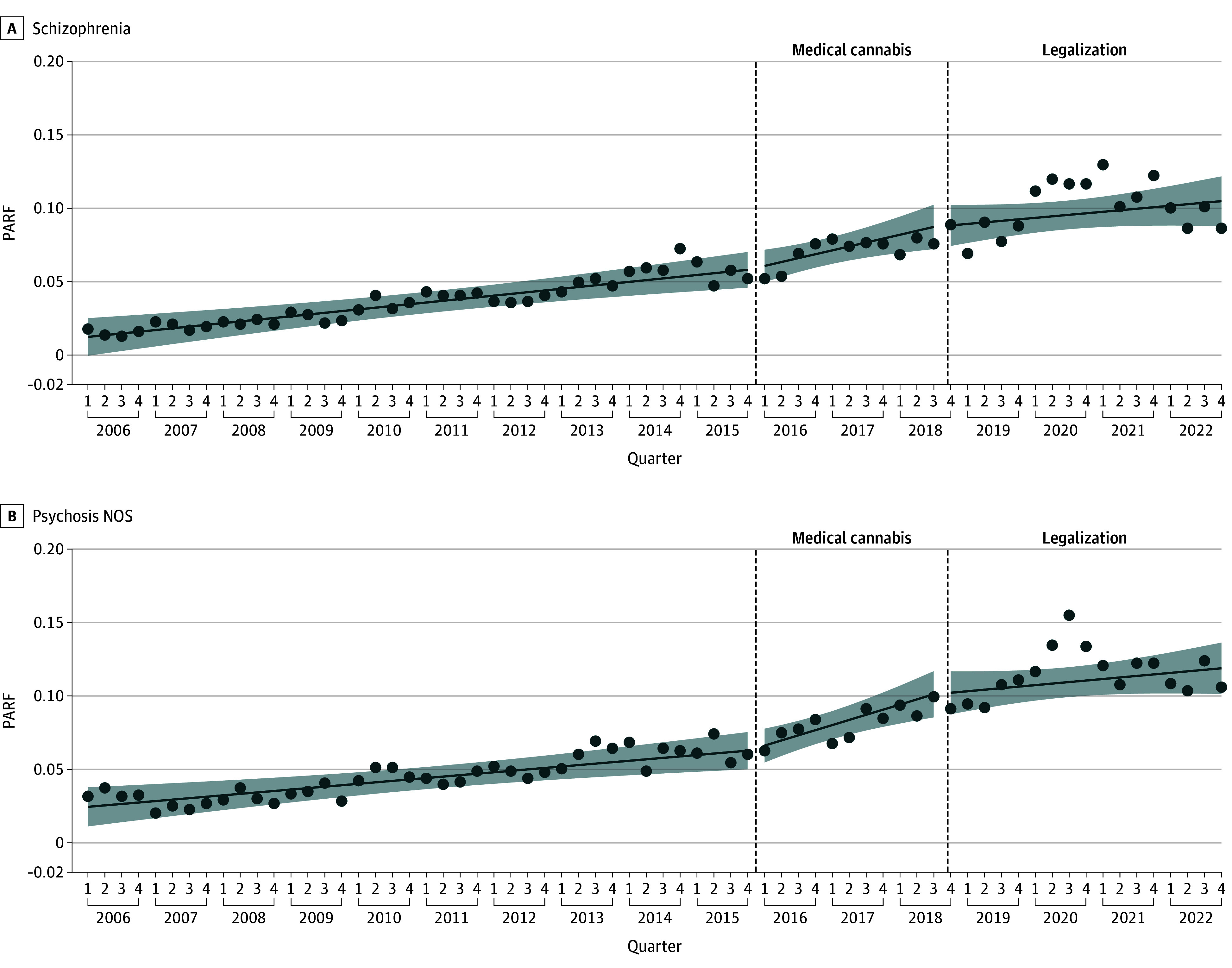
Changes in the Population-Attributable Fraction (PARF) for Cannabis Use Disorder Associated With Schizophrenia and Psychosis Not Otherwise Specified (NOS) PARF is shown as a proportion. The dashed vertical dashed lines indicate, from left to right, the liberalization of medical and nonmedical cannabis and the legalization of nonmedical cannabis. The dots indicate the observed quarterly PARF, and the line indicates the projected quarterly PARF (eg, model estimated value). Shaded regions indicate 95% CIs.

Figure 3 shows the interrupted time series model estimating the association of nonmedical cannabis liberalization in 2015 and legalization in 2018 with changes in the PARF for CUDs associated with schizophrenia and psychosis NOS. During the prelegalization period the PARF of CUD associated with schizophrenia was increasing significantly by 0.1% per quarter (95% CI, 0.1%-0.2%) and medical cannabis liberalization and nonmedical cannabis legalization with restrictions were not associated with any significant gradual changes in the prelegalization trend of an increasing PARF over time. During the prelegalization period, the PARF of CUD associated with psychosis NOS increased significantly by 0.1% per quarter (95% CI, 0.04%-0.2%). Medical cannabis liberalization was associated with a significant slope change, and the PARF increased by 0.3% per quarter (95% CI, 0.03%-0.5%). Nonmedical legalization with restrictions was not associated with any further significant changes in slope (see eTable 3 in Supplement 1 for full model coefficients).

## Discussion

Over the past 17 years, the proportion of new cases of schizophrenia in Ontario, Canada, associated with diagnosed CUD has increased from 1.6% in 2006 to 9.6% in 2022. The percentage of new cases of schizophrenia associated with CUD increased gradually over time and did not accelerate after the liberalization of medical cannabis or the legalization of nonmedical cannabis. Increases in the percentage of new cases of psychosis NOS associated with CUD accelerated after medical cannabis legalization. The risk of developing schizophrenia associated with CUD was fairly stable over time, and the increases in the PARF were associated primarily with an increasing prevalence of CUD in the population. There were large variations in the PARF by age and sex, with a much larger proportion of cases of schizophrenia associated with CUDs among youths, particularly among males.

The increasing PARF for CUD associated with schizophrenia are consistent with population-representative surveys on cannabis use in Ontario. Between 2006 and 2022, the percentage of Ontario residents aged 18 years or older who reported past-year cannabis use and patterns placing them at moderate or high risk of experiencing social problems or cannabis dependence increased by 145.5% (from 13.4% in 2006 to 32.9% in 2022) and 223.3% (from 6.0% in 2006 to 19.4% in 2022), respectively.^28^ The THC content of cannabis in North America has more than doubled over our study time frame, with more than 70% of legal dried cannabis sold in Ontario currently exceeding 20% THC.^29^ Consistent with increasing cannabis use and potency, ED visits in Ontario for cannabis-induced psychosis and CUD have increased over time.^24,29^ Our results, and those of others, highlight that the association between CUD and schizophrenia may be particularly elevated among younger males, in whom an estimated 18.9% of incident schizophrenia cases were associated with CUD by the end of the study.^30^

Specific to cannabis policy, we observed no change in the prelegalization trend of increasing PARF over time for our primary outcome after medical cannabis liberalization and nonmedical cannabis legalization with restrictions. The PARF of CUD associated with our secondary outcome (psychosis NOS) did accelerate after medical cannabis liberalization in 2015. These findings should be interpreted with 2 caveats. First, attribution of the associations of changes in cannabis policy from changing social norms and behaviors in the lead-up to changes is challenging. Although evidence from the US and Canada has found large increases over time in CUDs, the association of legalization with these trends has been modest, with increases also occurring in regions without legal cannabis.^24,31^ Prior work has supported that cannabis use in Canada began increasing prelegalization when the government announced it intended to legalize cannabis and there were increasing societal discussions and acceptance of cannabis.^32^ Second, our study does not fully evaluate cannabis legalization, as the market was heavily restricted before 2020, leaving less than 2 years of higher retail and high-potency product exposure for individuals to develop new CUDs, develop schizophrenia, and receive a formal diagnosis. The final 2 years of our study also overlapped with the COVID-19 pandemic, which resulted in changes in health service use. Evidence suggests that greater exposure to cannabis retail stores and higher-potency cannabis products is associated with increased cannabis use and harms and ongoing monitoring is indicated.^8,33,34^

The overall incidence of schizophrenia in our study was stable, consistent with prior work on changes immediately after cannabis legalization in Canada.^35^ However, this stability occurred because the incidence of schizophrenia increased among younger individuals while decreasing in older adults. Increasing rates of schizophrenia among younger adults are of considerable concern and may be capturing important harms associated with increasing cannabis use, as youths are considered the most vulnerable to cannabis use. Increases over time in PARF were much larger than the changes in incidence of schizophrenia. There are several potential explanations for this discrepancy. First, rates of schizophrenia may be subject to diagnostic drift, where clinicians have become less willing to diagnose individuals with schizophrenia due to increasing rates of concurrent substance use disorders. This possibility is supported by our findings that the incidence of psychosis NOS increased over our study time frame and accelerated after the legalization of medical cannabis. Second, part of the increase in PARF may capture better detection and documentation of CUD over time rather than an actual increase in the prevalence of CUD. In this case, more recent estimates are now better capturing the underlying burden of CUD on schizophrenia. Third, CUD may have a relatively small causal effect on the incidence of schizophrenia at the population level, with associations observed in our study possibly being due to confounding environmental and genetic factors.^36,37^

### Limitations

Our study has several limitations. First, our PARF estimations are considered valid only assuming all relevant confounders have been identified and adjusted for. We did not have access to several important potential confounders, including individual-level income, educational attainment, family history of mental health disorders, and genetics. Consequently, while the magnitude of our PARFs should be interpreted with caution (eg, diagnosed CUD may not cause 10% of cases of schizophrenia), the increases over time likely capture important trends. Second, our exposure definition of CUD (treatment in the ED or hospital) is specific but less sensitive for CUD. Individuals with CUD or who heavily use cannabis, who received outpatient care, or did not seek care would be misclassified in the general population, biasing our results toward the null. Estimates suggest approximately 2.3% of individuals in Ontario have had problems stopping or cutting back on cannabis use in the past 3 months, which is almost triple the prevalence of CUD treatment in our study.^38^ Third, although the exposure objectively captures a clinically relevant pattern of cannabis use requiring ED or hospital-based care, some of these individuals may not have CUD. Fourth, we used a 10-year lookback period to establish an incident diagnosis of schizophrenia, and individuals who had more than 10 years between episodes of care would be counted twice. This bias is consistent over time. Fifth, the final period of our study overlapped with the COVID-19 pandemic, during which time there were important impacts on health service use, possibly underestimating the number of incident cases with schizophrenia, which could influence the HRs and PARF estimates in this period. Ongoing research examining postpandemic periods and tracking changes as the cannabis market matures, including increasing THC content of products, is indicated.

## Conclusions

In this cohort study of individuals aged 14 to 65 years in Ontario, the proportion of incident cases of schizophrenia associated with CUD almost tripled during a period encompassing ongoing liberalization of medical and nonmedical cannabis. Although the proportion of cases of schizophrenia associated with CUD increased fairly linearly over time, incident cases of psychosis NOS and the proportion associated with CUD accelerated after cannabis liberalization. Ongoing research is needed on long-term trends in the incidence of psychotic disorders associated with changes in cannabis policy, particularly the commercialization of the legal cannabis market, which was not well captured in this study.

## Notes

Supplement 1. **eMethods 1.** Data Sources and Data Availability **eMethods 2.** Outcome Definition **eMethods 3.** Covariates
eReferences.
**eFigure.** Cohort Flow Chart **eTable 1.** Crude and Standardized Annual Rates of Incident SSD and Psychosis NOS **eTable 2.** Sensitivity Analyses for PARF of Cannabis Use Disorder Associated With Incident Schizophrenia Spectrum Disorder **eTable 3.** Interrupted Time Series Analyses of Absolute Changes in the PARF (Expressed as a Percentage) of Cannabis Use Disorder Associated With Schizophrenia and Psychosis Not Otherwise Specified (NOS)

Supplement 2.
Data Sharing Statement
