# Supplementary material for: Changes in Incident Schizophrenia Diagnoses Associated With Cannabis Use Disorder After Cannabis Legalization
Source: JAMA Netw Open. 2025 Feb 4;8(2):e2457868. doi: 10.1001/jamanetworkopen.2024.57868 (PMC11795325; doi:10.1001/jamanetworkopen.2024.57868)
Supplement: Supplement 1. — eMethods 1. Data Sources and Data Availability eMethods 2. Outcome Definition eMethods 3. Covariates eReferences. eFigure. Cohort Flow Chart eTable 1. Crude and Standardized Annual Rates of Incident SSD and Psychosis NOS eTable 2. Sensitivity Analyses for PARF of Cannabis Use Disorder Associated With Incident Schizophrenia Spectrum Disorder eTable 3. Interrupted Time Series Analyses of Absolute Changes in the PARF (Expressed as a Percentage) of Cannabis Use Disorder Associated With Schizophrenia and Psychosis Not Otherwise Specified (NOS) [file jamanetwopen-e2457868-s001.pdf]

Supplementary Online Content

Myran DT, Pugliese M, Harrison LD, et al. Changes in incident schizophrenia diagnoses associated with cannabis use disorder after cannabis legalization. *JAMA Netw Open.* 2025;8(2):e2457868. doi:10.1001/jamanetworkopen.2024.57868

eMethods 1. Data Sources
eMethods 2. Outcome Definition
eMethods 3. Covariates
eReferences.
eFigure. Cohort Flow Chart
eTable 1. Crude and Standardized Annual Rates of Incident SSD and Psychosis NOS
eTable 2. Sensitivity Analyses for PARF of Cannabis Use Disorder Associated With Incident Schizophrenia Spectrum Disorder
eTable 3. Interrupted Time Series Analyses of Absolute Changes in the PARF (Expressed as a Percentage) of Cannabis Use Disorder Associated With Schizophrenia and Psychosis Not Otherwise Specified (NOS)

This supplementary material has been provided by the authors to give readers additional information about their work.

© 2025 Myran DT et al. *JAMA Network Open.*

eMethods 1. Data Sources

We obtained study data from de-identified and linked health administrative databases housed at ICES. ICES is an independent, non-profit research institute funded by an annual grant from the Ontario Ministry of Health and Long-Term Care (MOHLTC). As a prescribed entity under Ontario's privacy legislation, ICES is authorized to collect and use health care data for the purposes of health system analysis, evaluation and decision support. Secure access to these data is governed by policies and procedures that are approved by the Information and Privacy Commissioner of Ontario. In 2018, the institute formerly known as the Institute for Clinical Evaluative Sciences formally adopted the initialism ICES as its official name.

The dataset from this study is held securely in coded form at ICES. While legal data sharing agreements between ICES and data providers (e.g., healthcare organizations and government) prohibit ICES from making the dataset publicly available, access may be granted to those who meet pre-specified criteria for confidential access, available at www.ices.on.ca/DAS (email: das@ices.on.ca). The full dataset creation plan and underlying analytic code are available from the authors upon request, understanding that the computer programs may rely upon coding templates or macros that are unique to ICES and are, therefore, either inaccessible or may require modification.

These datasets were linked using unique encoded identifiers and analyzed at ICES.
We used the following databases:

- National Ambulatory Care Reporting System (NACRS), which captures all ED visits and the cause of the visit within Ontario;
- Discharge Abstract Database (DAD), which includes records for all acute care hospitalizations in Ontario,
- Ontario Mental Health Reporting System Metadata (OMHRS) which includes all mental health hospitalizations in Ontario,
- OHIP Claims Database (OHIP, which captures all outpatient visits (including virtual) and the reason for visit in Ontario;
- Registered Persons Database (RPDB), which includes the total number of persons at-risk each month and individuals' age and sex; and
- Postal Code Conversation File+ (PCCF+) which contains information on the rurality (urban vs rural) and neighbourhood income for each person's home address.
- CIC-IRCC Permanent Residents database, which includes records for all landed immigrants, refugees, etc. in Ontario from January 1985 to September 2020. The end date is updated as new data are made available.

© 2025 Myran DT et al. *JAMA Network Open.*

eMethods 2. Outcome Definition

We defined an incident diagnosis of schizophrenia spectrum disorder (SSD) as an individual with no outpatient, ED, or hospital-based care for SSD or substance-induced psychosis in the past 3-years who met one or more of the following 3 criteria after an incident ED visit caused by hallucinogen use or after their assigned pseudo index date for members of the general population.

1) ICD-10 code (F20x or F25x) for a primary discharge diagnosis of schizophrenia or schizoaffective disorder from a general hospital bed; or
2) A DSM-IV diagnostic code (295x) or ICD-10 code (F20x or F25x) for a diagnosis of schizophrenia or schizoaffective disorder from a psychiatric hospital bed; or
3) Two or more outpatient OHIP billing claims (ICD-9 code 295.x) or ED visits with a diagnostic code for schizophrenia or schizoaffective disorder (ICD-10 code F20x or F25x) within a 12-month period.

These criteria were informed by chart reviewed validated coding (sensitivity 91.6%, specificity of 61.3%) for diagnosis of schizophrenia/schizoaffective disorder).(1)

© 2025 Myran DT et al. *JAMA Network Open.*

eMethods 3. Covariates

We obtained sociodemographic details including age, sex (male or female), rural residence (urban, rural or missing), neighborhood income quintile (Q1-5, and missing) and recent immigration status (arrived in Canada between 1985 and 2022). Urban or rural residence and neighborhood income level quintiles were defined using Statistics Canada census data.(2) Recent immigration status was defined using the Immigration, Refugees and Citizenship Canada Permanent Resident database.(3) Using standardized coding we obtained information on healthcare use in the past 3 years, including outpatient mental health visits (family medicine, or psychiatry) and ED visits and hospitalizations for substance use (alcohol, cannabis, cocaine, polysubstance use, amphetamine use, and other) and mental health conditions (mood disorder, anxiety disorder, self-harm, and other).(4,5)

© 2025 Myran DT et al. *JAMA Network Open.*

eReferences.

1. Kurdyak P, Lin E, Green D, Vigod S. Validation of a Population-Based Algorithm to Detect Chronic Psychotic Illness. Can J Psychiatry [Internet]. 2015; Aug 1;60(8):362–8. Available at: https://pubmed.ncbi.nlm.nih.gov/26454558/
2. Statistics Canada. Population Centre and Rural Area Classification. 2016.
3. Chiu M, Lebenbaum M, Lam K, Chong N, Azimaee M, Iron K, Manuel D, Guttmann A. *BMC Med Infrom Decis Mak*. 2016; 16(1):135. Epub 2016 Oct 21. Available at: https://bmcmedinformdecismak.biomedcentral.com/articles/10.1186/s12911-016-0375-3
4. MHASEF Research Team. Mental Health and Addictions System Performance in Ontario: A Baseline Scorecard [Internet]. Available at: https://www.ices.on.ca/Publications/Atlases-and-Reports/2018/MHASEF; 2018.
5. Chiu M, Guttmann A, Kurdyak P. Mental Health and Addictions System Performance in Ontario: An Updated Scorecard, 2009-2017. Healthc Q [Internet]. 2020 Oct 1 [cited 2023 Jan 31];23(3):7–11. Available from: https://pubmed.ncbi.nlm.nih.gov/33243359/

© 2025 Myran DT et al. *JAMA Network Open.*

eFigure. Cohort Flow Chart

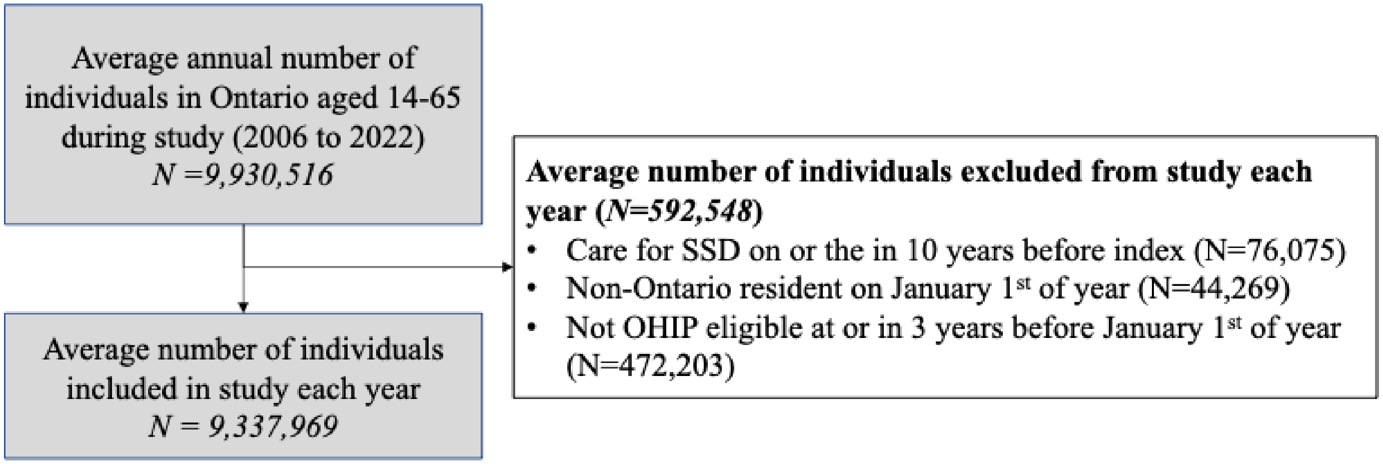

© 2025 Myran DT et al. *JAMA Network Open.*

eTable 1. Crude and Standardized Annual Rates of Incident SSD and Psychosis NOS

	Schizophrenia Spectrum Disorder			Psychosis Not Otherwise Specified		
	Crude	Age-Sex Standardized	Age-Sex, Rural, Immigration Status Standardized	Crude	Age-Sex Standardized	Age-Sex, Rural, Immigration Status Standardized
**Year**	**Annual incidence per 100,000 Individuals (95%CI)**					
2006	69.20	69.33 (67.58 - 71.07)	68.04 (66.27 - 69.82)	30.49	29.98 (28.84 - 31.11)	29.68 (28.47 - 30.88)
2007	61.44	61.35 (59.70 - 62.99)	60.44 (58.74 - 62.13)	31.20	30.66 (29.51 - 31.82)	30.24 (29.04 - 31.45)
2008	59.18	59.05 (57.44 - 60.66)	58.12 (56.48 - 59.76)	31.35	30.80 (29.65 - 31.96)	30.20 (29.04 - 31.37)
2009	59.10	59.03 (57.43 - 60.63)	58.34 (56.73 - 59.95)	31.70	31.18 (30.02 - 32.33)	30.80 (29.64 - 31.96)
2010	56.97	56.87 (55.31 - 58.43)	58.13 (54.29 - 61.96)	33.13	32.55 (31.38 - 33.73)	32.32 (31.13 - 33.50)
2011	56.34	56.14 (54.61 - 57.68)	55.84 (54.30 - 57.39)	35.76	35.05 (33.85 - 36.26)	34.79 (33.58 - 36.00)
2012	55.33	55.16 (53.65 - 56.67)	54.62 (53.11 - 56.13)	36.44	35.82 (34.61 - 37.03)	35.32 (34.11 - 36.52)
2013	57.10	56.87 (55.35 - 58.40)	61.30 (54.74 - 67.86)	39.72	39.04 (37.78 - 40.30)	38.79 (37.53 - 40.05)
2014	53.07	52.86 (51.39 - 54.33)	52.70 (51.23 - 54.17)	41.01	40.34 (39.06 - 41.61)	40.16 (38.88 - 41.43)
2015	52.54	52.56 (51.10 - 54.03)	52.44 (50.97 - 53.90)	40.71	40.37 (39.09 - 41.65)	40.14 (38.86 - 41.41)
2016	52.49	52.51 (51.05 - 53.96)	52.22 (50.77 - 53.68)	42.60	42.38 (41.07 - 43.69)	42.11 (40.81 - 43.41)
2017	53.35	53.53 (52.06 - 55.00)	53.29 (51.82 - 54.75)	46.64	46.57 (45.20 - 47.94)	46.34 (44.97 - 47.70)
2018	55.51	55.76 (54.27 - 57.25)	55.47 (53.98 - 56.96)	46.53	46.79 (45.42 - 48.16)	46.48 (45.12 - 47.85)
2019	55.32	55.79 (54.29 - 57.28)	55.61 (54.12 - 57.09)	45.84	46.21 (44.85 - 47.57)	46.01 (44.66 - 47.37)
2020	53.68	54.18 (52.70 - 55.66)	54.12 (52.65 - 55.60)	52.77	53.32 (51.85 - 54.79)	53.18 (51.72 - 54.65)
2021	54.63	55.19 (53.71 - 56.67)	55.21 (53.72 - 56.69)	59.90	60.49 (58.94 - 62.05)	60.42 (58.86 - 61.97)
2022	53.98	54.51 (53.04 - 55.97)	54.55 (53.08 - 56.02)	54.63	55.09 (53.61 - 56.56)	55.05 (53.58 - 56.53)

© 2025 Myran DT et al. *JAMA Network Open.*

eTable 2. Sensitivity Analyses for PARF of Cannabis Use Disorder Associated With Incident Schizophrenia Spectrum Disorder

Analysis Year	Unadjusted with 10 year exclusion lookback	Adjusted for Age, Sex, Income, Rurality and immigration Status with 10 year exclusion lookback	Fully Adjusted with 10 year exclusion lookback*	Fully Adjusted with unlimited exclusion lookback
		PARF (95%CI)		
2006	0.0348 (0.0309-0.0387)	0.0347 (0.0308-0.0386)	0.0158 (0.0114-0.0203)	0.0169 (0.0123-0.0216)
2007	0.0392 (0.0351-0.0432)	0.0389 (0.0349-0.0430)	0.0206 (0.0159-0.0252)	0.0219 (0.0171-0.0268)
2008	0.0401 (0.0361-0.0441)	0.0398 (0.0359-0.0438)	0.0223 (0.0177-0.0270)	0.0226 (0.0178-0.0274)
2009	0.0459 (0.0417-0.0502)	0.0457 (0.0414-0.0499)	0.0273 (0.0225-0.0322)	0.0293 (0.0242-0.0343)
2010	0.0544 (0.0499-0.0589)	0.0541 (0.0496-0.0586)	0.0366 (0.0316-0.0416)	0.0395 (0.0343-0.0448)
2011	0.0621 (0.0575-0.0667)	0.0618 (0.0572-0.0664)	0.0431 (0.0380-0.0482)	0.0456 (0.0403-0.0510)
2012	0.0607 (0.0564-0.0650)	0.0603 (0.0560-0.0646)	0.0386 (0.0335-0.0437)	0.0402 (0.0348-0.0455)
2013	0.0735 (0.0690-0.0779)	0.0730 (0.0686-0.0775)	0.0493 (0.0440-0.0546)	0.0516 (0.0461-0.0572)
2014	0.0903 (0.0857-0.0950)	0.0898 (0.0851-0.0944)	0.0628 (0.0570-0.0685)	0.0677 (0.0616-0.0738)
2015	0.0883 (0.0839-0.0927)	0.0876 (0.0832-0.0920)	0.0570 (0.0511-0.0628)	0.0629 (0.0567-0.0691)
2016	0.0991 (0.0947-0.1034)	0.0983 (0.0939-0.1027)	0.0642 (0.0582-0.0702)	0.0685 (0.0621-0.0750)
2017	0.1176 (0.1132-0.1220)	0.1167 (0.1123-0.1210)	0.0790 (0.0727-0.0853)	0.0835 (0.0767-0.0903)
2018	0.1217 (0.1176-0.1258)	0.1207 (0.1166-0.1248)	0.0804 (0.0742-0.0867)	0.0868 (0.0801-0.0936)
2019	0.1298 (0.1259-0.1336)	0.1286 (0.1247-0.1325)	0.0838 (0.0773-0.0904)	0.0912 (0.0841-0.0983)
2020	0.1611 (0.1570-0.1652)	0.1599 (0.1558-0.1640)	0.1195 (0.1131-0.1260)	0.1325 (0.1255-0.1395)
2021	0.1638 (0.1598-0.1678)	0.1625 (0.1584-0.1665)	0.1176 (0.1108-0.1244)	0.1285 (0.1212-0.1358)
2022	0.1528 (0.1489-0.1567)	0.1514 (0.1476-0.1553)	0.0956 (0.0880-0.1032)	0.1015 (0.0932-0.1098)

Numbers are proportions.

**This is the model of the primary analyses.*

© 2025 Myran DT et al. *JAMA Network Open.*

eTable 3. Interrupted Time Series Analyses of Absolute Changes in the PARF (Expressed as a Percentage) of Cannabis Use Disorder Associated With Schizophrenia and Psychosis Not Otherwise Specified (NOS)

	**Schizophrenia**	**Psychosis NOS**
	PARF (95%CI)	
**Intercept**	1.32	2.28
**Pre-legalization**		
Overall Quarterly Slope	0.12 (0.07-0.17)	0.10 (0.04-0.15)
**Medical Cannabis period**		
Gradual Quarterly Change	0.15 (-0.07-0.36)	0.25 (0.03-0.48)
Overall Quarterly Slope	0.27 (0.09-0.44)	0.35 (0.16-0.54)
**Legalization Period**		
Gradual Quarterly Change	-0.16 (-0.46-0.14)	-0.24 (-0.56-0.07)
Overall Quarterly Slope	0.10 (-0.45-0.65)	0.10 (-0.47-0.68)

The PARF is expressed as a percentage and changes are expressed in absolute changes. For example, pre-legalization the PARF for CUD on schizophrenia was increasing by 0.12% per quarter.

© 2025 Myran DT et al. *JAMA Network Open.*
